# Having a Family Doctor is Associated with Some Better Patient-Reported Outcomes of Primary Care Consultations

**DOI:** 10.3389/fmed.2014.00029

**Published:** 2014-09-15

**Authors:** Cindy L. K. Lam, Esther Y. T. Yu, Yvonne Y. C. Lo, Carlos K. H. Wong, Stewart M. Mercer, Daniel Y. T. Fong, Albert Lee, Tai Pong Lam, Gabriel M. Leung

**Affiliations:** ^1^Department of Family Medicine and Primary Care, The University of Hong Kong, Hong Kong, China; ^2^Section of General Practice and Primary Care, Institute of Health and Wellbeing, University of Glasgow, Glasgow, UK; ^3^School of Nursing, The University of Hong Kong, Hong Kong, China; ^4^Centre for Health Education and Health Promotion, The Jockey Club School of Public Health and Primary Care, The Chinese University of Hong Kong, Hong Kong, China; ^5^School of Public Health, The University of Hong Kong, Hong Kong, China

**Keywords:** patient-reported outcomes, patient-centered care, patient enablement, family doctor, primary care

## Abstract

**Background:** Hong Kong (HK) has pluralistic primary care that is provided by a variety of doctors. The aim of our study was to assess patient-reported outcomes of primary care consultations in HK and whether having a family doctor (FD) made any difference.

**Methods:** We interviewed by telephone 3148 subjects from 5174 contacted households (response rate 60.8%) randomly selected from the general population of HK about the experience of their last primary care consultations in September 2007 and April 2008. We compared the patient-reported outcomes (PRO) and patient-centered process of care in those with a FD, those with other types of regular primary care doctors (ORD) and those without any regular primary care doctor (NRD). PRO included patient enablement, global improvement in health, overall satisfaction, and likelihood of recommending their doctors to family and friends. Patient-centered process of care indicators was explanations about the illness, and address of patient’s concerns.

**Results:** One thousand one hundred fifty, 746, and 1157 reported to have FD, ORD, and NRD, respectively. Over 80% of those with FD consulted their usual primary care doctors in the last consultation compared with 27% of those with NRD. Compared with subjects having ORD or NRD, subjects with FD reported being more enabled after the consultation and were more likely to recommend their doctors to family and friends. Subjects with FD and ORD were more likely than those having NRD to report a global improvement in health and satisfaction. FD group was more likely than the other two groups to report receiving an explanation on the diagnosis, nature, and expected course of the illness, and having their concerns addressed. Patient enablement was associated with explanation of diagnosis, nature, and expected course of illness, and address of patient’s concerns.

**Conclusion:** People with a regular FD were more likely to feel being enabled and to experience patient-centered care in consultations.

## Introduction

Primary care is the first contact of care that is expected to manage up to 90% of health problems of the population (1, 2). The outcomes of primary care consultations have an important impact on the health of the population and workload of secondary and tertiary care. Traditional clinical outcomes such as mortality or disability lack sensitivity as quality indicators of primary care because the majority of the problems encountered are functional or self-limiting (3). New concepts and outcome measures have been developed to assess the quality of primary care (4, 5). Patient-centered care is one of the key concepts (6–8) and patient-reported outcomes (PRO) are the key measures of the effectiveness and quality of clinical practice in the primary care setting (9). In general terms, patient-centered care is about the understanding of the patient’s thoughts, feelings, and expectations in the context of their illnesses in order to reach a common ground for integrated management (10). An important goal of primary care consultations is to enhance patient enablement and satisfaction, promote engagement and task orientation, and lessen anxiety and improve health (11–13). Little et al. showed that a positive and clear approach in the consultation was an independent predictor of patient enablement and satisfaction (14).

Starfield et al. found that countries with a more uniform type of primary care providers have better population health outcomes (1). Some studies have shown that increased family physician supply, but not other types of primary care providers, was linked with improved health outcomes (15, 16). Most of the data on outcomes of primary care consultations had come from Western populations and were based on cross-nation comparisons (1, 15, 16). Little is known about the outcomes of consultations perceived by individual patients using different types of doctors in a pluralistic primary care system.

In Hong Kong (HK), similar to the United States and many other Asian countries, primary care is provided by a variety of doctors with variable duration or background of postgraduate training, ranging from general practitioners with only a basic medical degree to specialists in family medicine or other disciplines. Although vocational training in family medicine and formal examinations have been available for more than 20 years, it is not mandatory for independent practice in primary care in HK. Around 80% of the primary care consultations are funded privately (17) and insurance covers only 27% of the private health expenditure (18). There is a free market where people pay for their medical services and are free to choose their doctors. There was some concern about the effectiveness of such fragmented primary care in HK and the Government initiated a Health Care Reform to promote the family doctor (FD) concept for continuous, comprehensive and patient-centered primary care in 2005 (19). The question is whether having a FD really makes any difference to the quality of primary care to the person.

The aim of our study was to assess PRO of consultations in the pluralistic primary care setting in HK, and whether having a FD made any difference. The PRO indicators included patient enablement (20), subjective perception of global improvement in health and satisfaction. This study compared these PRO of consultations among patients using different types of primary care providers and explored their associations with the patient-centered process of consultation in terms of perceived explanation of illness and address of concerns.

## Materials and Methods

### Study design and setting

A random telephone survey on utilization patterns and outcomes of primary care consultations on the general population of HK was carried out by two phases, to reduce seasonal bias, in September 2007 and April 2008. Telephone-owning households were contacted by random digital dialing using a computer assisted telephone inventory system that had a 95% household coverage rate, conducted by the Social Science Research Centre of the University of Hong Kong. A member of the household who would next have a birthday was recruited for the survey. We used a structured questionnaire to collect data on socio-demographics, chronic morbidity, choice of primary care providers, service utilization patterns, and the patient-reported process and outcomes of the last primary care consultation (Supplementary Material). All adults answered the questionnaire in person and the main carer of children under the age of 18 answered the survey as the proxy. Details of the study methods were described in previous papers (17, 21). The study was approved by the Institutional Review Board of the University of Hong Kong/Hospital Authority Hong Kong West Cluster (HKU/HA HKW IRB Ref number UW 07-021).

### Definition of study groups

In our study, we defined a primary care doctor as one whom an individual would first consult when he/she needs medical care and a FD as one whom he/she would first consult and for all types of health problems. The identification of a “FD” was based on the perception of the subjects regarding the comprehensiveness of care, which differentiated FDs from other types of providers in the pluralistic primary care system. We first asked the respondent whether he or she had a regular primary care doctor (whom you usually would first consult when you need to see a doctor), and then we asked the respondent whether he or she thought his or regular primary care doctor was a FD (whom he or she would consult for all his or her health problems). We classified the subjects into three comparison groups: (1) those having a FD; (2) those having other types of regular primary care doctors (ORD), who can be any doctor including specialists in any discipline or even a traditional Chinese medicine practitioner but the subjects did not consider them as their FDs; and (3) those with no regular primary care doctors (NRD).

### Measures of patient-reported outcomes and process the consultation

We used the Chinese version of the patient enablement instrument (PEI) (22) to measure patient enablement. The PEI was originally developed in the United Kingdom (UK) by Howie et al. (3, 23). It was translated and validated for the HK Chinese population (22). It had also been used in other populations to be a valid and sensitive PRO measure of the quality of primary care consultations (23–26). The PEI has six items that measure the change in the patient’s perceived: (i) ability to cope with life; (ii) ability to understood one’s illness; (iii) ability to cope with one’s illness; (iv) ability to keep oneself healthy; (v) confidence about one’s health; and (iv) ability to help oneself, after the doctor’s consultations. Each item is scored on four response options: “the same or less” (0), “slightly improved/increased” (1), “greatly improved/increased” (2), or “not applicable.” The PEI score was calculated as the mean of applicable items multiplied by six if three or more items are applicable. Cases that had more than three items being marked as “not applicable” or with missing items were excluded from the analysis.

We asked each subject to rate their global change in health after the last consultations with the global rating scale (GRS) (27, 28) that consisted of a 7-point ordinal scale from much better (+3) to much worse (−3). The GRS had demonstrated good sensitivity in many other studies in the HK Chinese population (29–31).

Patient satisfaction was measured by two items, one on overall satisfaction and one on the likelihood of recommending the doctor to family and friends. Responses were rated on a 5-point Likert scale. These are generic questions that can provide a composite evaluation of different aspects of the consultation (32). Previous studies showed that there was correlation between overall satisfaction and various domains of visit-specific satisfaction (33–35). The likelihood of recommendation of his/her doctor to others implies the patient has confidence in the doctor and acknowledges the quality of care (36). The data of each item were analyzed separately.

We measured the process of patient-centered care using one question each to assess whether the diagnosis, nature, and expected course of illness had been explained and whether concerns had been addressed and reassured in the consultation. Responses included “yes,” “no,” and “not sure.” Studies have shown that providing information and explaining about the condition to patients helped to achieve mutual understanding of the problem and facilitate patient involvement in decision-making (37, 38).

### Data analysis

The mean PEI score and the proportions of people who were enabled (PEI > 0) overall and by groups were calculated. For the question on GRS, we dichotomized the responses by treating “much better/better/a little better” as “better,” and “same/a little worse/worse/much worse” as “no better.” Similarly, responses to the question on patient overall satisfaction were dichotomized into “satisfied” and “not satisfied.” For the question on recommending the doctors to family and friends, we dichotomized the responses by treating “definitely yes/maybe yes” as “yes,” and “not sure/maybe not/definitely not” as “no.” We performed multiple logistic or ordinary linear regression analyses to determine the effect of having a FD on each of the patient-reported process and outcome indicators of the consultation. Model adequacy of logistic regressions was indicated by Hosmer–Lemeshow test and that of linear regressions was indicated by normality of residuals and random pattern on the scatter plot of residuals against predicted values. The analyses were adjusted for potential confounding variables including age, sex, occupation, marital status, household monthly income, education level, district of living, a single-item subjective rating of general health status, presence of chronic diseases, need for long-term medication, smoking and drinking habits, and regular exercise. We also explored the relationships between the reported consultation processes and each of the PRO using logistic regression and ordinary linear regression analyses. Data were analyzed using the SPSS version 20.0. *P*-values of <0.05 were considered statistically significant.

## Results

A total of 3148 subjects from 5174 contacted households completed the survey (1616 subjects and 1532 subjects in the first and second sampling phases, respectively). The overall response rate was 60.8%. Among the 3148 subjects, 1150 (36.5%) reported having a FD, 746 (23.7%) said they had other types of regular primary care doctors (ORD), and 1157 (36.8%) did not have any regular primary care doctors (NRD). Ninety-five subjects (3%) were not sure whether they had any regular primary care doctor or FD.

Table 1 shows the socio-demographic characteristics of all subjects and by groups. The mean age of the FD group was significantly younger than that of the NRD group. The FD group was more likely than the NRD group to have higher household incomes. The general health condition tended to be better in the FD than the ORD group. There was no statistical difference among the three groups in terms of education, occupation, having chronic disease, and taking long-term medications.

**Table 1:** **Socio-demographics of subjects by primary care doctor choice groups**.

	FD^f^	ORD^f^	NRD^f^	Overall^f^
	(*n* = 1150)	(*n* = 746)	(*n* = 1157)	(*n* = 3148)
Age (mean ± SD)^a^	38.5 ± 17.6	40.1 ± 18.0	41.3 ± 18.5	40.2 ± 18.1
<25 (%)	21.9	23.8	24.1	23.0
25–64 (%)	72.8	67.4	63.9	68.0
≥65 (%)	5.3	8.7	12.0	9.0
Male (%)	38.1	39.4	44.9	40.9
Married (%)	57.1	53.6	53.2	54.9
Education ≤ primary school (%)	13.4	17.1	20.7	17.4
Household income (%)^b^^,^^c^
<$10,000^b^	14.4	25.2	32.1	24.3
$10,001–$19,999	27.6	26.8	32.0	28.8
$20,000–$29,999	20.5	21.0	14.9	18.3
$30,000–$39,999	12.5	9.5	8.6	10.3
≥$40,000^b^	25.0	17.5	12.5	18.3
Occupation (%)
Blue collar/service sales worker	17.4	21.7	23.0	20.3
Managerial/admin/professional/employer	16.0	12.3	9.7	12.5
White collar workers	19.5	17.5	13.0	16.5
Chronic disease (%)	34.8	38.2	30.8	34.7
Long-term medications (%)	23.7	25.1	19.5	22.8
Consultations past 4 weeks (mean ± SD)^a^^,^^e^	0.85 ± 1.73	0.85 ± 1.60	0.49 ± 1.26	0.7 ± 1.53
General health condition (%)^d^
Excellent/very good/good	53.2	41.3	50.0	48.7
Fair	42.3	49.2	44.3	44.9
Poor	4.3	9.5	5.8	6.4

*SD, standard deviation; FD, family doctor; ORD, other types of regular primary care doctor; NRD, no regular primary care doctor*.

*^a^Significant difference between FD and NRD by least square difference (LSD) in one way ANOVA*.

*^b^Significant difference between FD and NRD by chi-square test*.

*^c^Median monthly domestic household income of HK population = HK$ 17,250 (39)*.

*^d^Significant difference between FD and ORD by chi-square test*.

*^e^Significant difference between FD and ORD by least square difference (LSD) in one way ANOVA*.

*^f^The sum of three groups did not add up to total as some respondents were not sure if they had regular/family doctors*.

### Outcomes and process of last consultations

Nine hundred thirty-two (81.0%) of the subjects in the FD group consulted their usual primary care doctors in the last consultation compared with 517 (69.3%) in the ORD and 309 (26.7%) in the NRD group. Table 2 shows the numbers and percentages of subjects in each group reporting on each of the PRO and process of care indicators of their last consultations. Overall, 1914 (67.4%) people felt more enabled (PEI score > 0) and 1544 (49.3%) perceived a global improvement in their health after their last consultations. The group with FD had the best results in all PRO and patient-centered process of care and the NRD group had the worst.

**Table 2:** **Patient-reported process and outcomes of consultation by primary care doctor choice groups**.

*N* = 3148	FD	ORD	NRD	Overall
	(*n* = 1150)	(*n* = 746)	(*n* = 1157)	(*n* = 3148)
**Consulted the usual doctor (%)^a–c^**	932 (81.0)	517 (69.3)	309 (26.7)	1815 (57.7)
**Outcomes of care**
PEI score (mean ± SD)	3.33 ± 3.24	2.63 ± 2.87	2.58 ± 2.83	2.89 ± 3.02
Felt enabled (PEI score >0) (%)^a,c^	747 (70.7)	435 (65.2)	680 (65.4)	1914 (67.4)
Health improved (%)^a,b^	614 (53.5)	370 (50.0)	516 (44.8)	1544 (49.3)
Overall satisfied (%)^a,c^	1100 (96.1)	699 (93.8)	1057 (92.0)	2940 (93.8)
Would recommend doctor (%)^a–c^	875 (76.1)	455 (61.1)	509 (44.1)	1888 (60.1)
**Patient-centered care indicators**
Diagnosis explained (%)^a–c^	921 (80.1)	545 (73.1)	733 (63.4)	2267 (72.0)
Nature of illness explained (%)^a–c^	799 (69.5)	473 (63.4)	631 (54.5)	1959 (62.2)
Expected course of illness explained (%)^a–c^	558 (48.5)	298 (39.9)	399 (34.5)	1291 (41.0)
Concerns addressed (%)^a–c^	473 (41.1)	252 (33.8)	301 (26.0)	1052 (33.4)

*SD, standard deviation*.

*^a^Significant difference between FD and NRD by univariate logistic regression*.

*^b^Significant difference between ORD and NRD by univariate logistic regression*.

*^c^Significant difference between FD and ORD by univariate logistic regression*.

*PEI, patient enablement instrument, mean score calculated as mean of answered items times 6, excluding cases that answered N/A or missing in >3 items*.

The results of the difference in each of the PRO and processes of care indicators among the three groups tested by logistic regressions, both unadjusted and adjusted for confounding variables, are shown in Tables 3 and 4, respectively. The validity of logistic regressions reported in Tables 3–5 were supported by Hosmer–Lemeshow test (*P* > 0.05 for all). Table 3 shows that the mean PEI score was significantly greater in the FD group than in other groups, but there was no significant difference in PEI score between the ORD and NRD groups. Both FD and ORD groups were more likely than the NRD group to report a global improvement in health after the last primary care consultation, being satisfied and willing to recommend their doctors to family and friends. Although there was no significant difference between the FD and ORD groups in terms of global improvement in health and overall satisfaction with the last consultation, the former was significantly more likely to recommend their doctors to others.

**Table 3:** **Effects of primary care doctor choice group on PRO of consultation**.

*N* = 3148	FD vs. ORD^a^	FD vs. NRD^a^	ORD vs. NRD^a^
**Coefficient by ordinary linear regression**	**Effect (95% CI)**		
**PEI score**
Unadjusted	0.70* (0.41–0.99)	0.74* (0.49–1.00)	0.04 (−0.25–0.33)
Adjusted^b^	0.63* (0.34–0.92)	0.73* (0.47–0.99)	0.10 (−0.19–0.40)
**Coefficient by logistic regression**	**Odds ratio (95% CI)**		
**Felt enabled (PEI score >0)**
Unadjusted	1.29* (1.05–1.59)	1.28* (1.06–1.53)	0.99 (0.81–1.21)
Adjusted^b^	1.27* (1.03–1.57)	1.25* (1.04–1.51)	0.99 (0.80–1.21)
**Health improved**
Unadjusted	1.15 (0.96–1.39)	1.42* (1.21–1.67)	1.23* (1.02–1.48)
Adjusted^b^	1.15 (0.95–1.38)	1.40* (1.18–1.65)	1.22* (1.01–1.47)
**Overall satisfied**
Unadjusted	1.61* (1.06–2.45)	2.13* (1.48–3.07)	1.32 (0.92–1.91)
Adjusted^b^	1.29 (0.83–2.00)	2.00* (1.38–2.91)	1.55* (1.05–2.28)
**Would recommend doctor**
Unadjusted	2.03* (1.66–2.48)	4.03* (3.37–4.81)	1.99* (1.65–2.40)
Adjusted^b^	1.88* (1.52–2.32)	3.86* (3.19–4.66)	2.05* (1.68–2.51)

*Statistically significant, *P* < 0.05.

*^a^As reference category, odds ratio = 1*.

*^b^Adjustment of confounding factors including demographics, SF-12 PCS and MCS scores, chronic morbidity, lifestyle, and health status*.

*PEI, patient enablement instrument, mean score calculated as mean of answered items times 6, excluding cases that answered N/A or missing in >3 items*.

**Table 4:** **Effects of primary care doctor choice group on patient-centered process of consultation**.

*N* = 3148	FD vs. ORD^a^	FD vs. NRD^a^	ORD vs. NRD^a^
	
	Odds ratio (95% CI) by logistic regressions		
Diagnosis explained			
Unadjusted	1.48* (1.19, 1.84)	2.33* (1.93, 2.81)	1.57*(1.28, 1.92)
Adjusted^b^	1.40* (1.12, 1.75)	2.08* (1.71, 2.52)	1.48* (1.21, 1.82)
Nature of illness explained			
Unadjusted	1.31* (1.08, 1.60)	1.90* (1.60, 2.25)	1.44* (1.20, 1.74)
Adjusted^b^	1.24* (1.02, 1.52)	1.76* (1.47, 2.10)	1.42* (1.17, 1.72)
Expected course of illness explained			
Unadjusted	1.42* (1.18, 1.71)	1.79* (1.51, 2.12)	1.26* (1.05, 1.53)
Adjusted^b^	1.35* (1.12, 1.64)	1.69* (1.43, 2.01)	1.25* (1.03, 1.52)
Concerns addressed			
Unadjusted	1.37* (1.13, 1.66)	1.99* (1.67, 2.37)	1.45* (1.19, 1.77)
Adjusted^b^	1.35* (1.11, 1.64)	1.96* (1.64, 2.36)	1.46* (1.19, 1.79)

*Statistically significant, *P* < 0.05.

*^a^As reference category, odds ratio = 1*.

*^b^Adjustment of confounding factors including demographics, SF-12 health survey, chronic morbidity, lifestyle, and health status*.

**Table 5:** **Relationships between patient-centered process of care and PRO of consultation**.

	Ordinary linear regression	Logistic regression (independent variable, yes vs. no^a^)			
	PEI score	Felt enabled	Health improved	Overall satisfied	Would recommend doctor
	
	Effect (95% CI)	Odds ratio (95% CI)			
Diagnosis explained					
Unadjusted	0.67* (0.43, 0.92)	1.46* (1.23, 1.73)	1.29* (1.10, 1.51)	3.18* (2.37, 4.26)	2.54* (2.17, 2.98)
Adjusted^b^	0.59* (0.34, 0.84)	1.44* (1.21, 1.72)	1.21* (1.03, 1.42)	3.05* (2.24, 4.16)	2.04* (1.72, 2.42)
Nature of the illness explained					
Unadjusted	1.00* (0.77, 1.23)	1.95* (1.66, 2.29)	1.47* (1.27, 1.70)	3.08* (2.28, 4.17)	2.39* (2.06, 2.78)
Adjusted^b^	0.95* (0.72, 1.18)	1.91* (1.62, 2.26)	1.38* (1.19, 1.60)	2.98* (2.17, 4.09)	2.03* (1.73, 2.39)
Expected course of illness explained					
Unadjusted	1.28* (1.06, 1.50)	2.09* (1.77, 2.45)	1.53* (1.33, 1.77)	3.14* (2.18, 4.53)	2.12* (1.82, 2.46)
Adjusted^b^	1.25* (1.02, 1.47)	2.06* (1.74, 2.45)	1.46* (1.26, 1.69)	2.92* (2.00, 4.25)	1.87* (1.59, 2.20)
Concerns addressed					
Unadjusted	1.37* (1.15, 1.60)	2.58* (2.15, 3.09)	1.73* (1.49, 2.02)	3.46* (2.27, 5.26)	2.60* (2.21, 3.06)
Adjusted^b^	1.33* (1.10, 1.56)	2.55* (2.12, 3.08)	1.61* (1.38, 1.88)	3.21* (2.08, 4.95)	2.31* (1.94, 2.75)

*Statistically significant (*P* < 0.05).

*^a^As reference category; odds ratio = 1*.

*^b^Adjustment of confounding factors including doctor groups, demographics, SF-12 health survey, chronic morbidity, lifestyle, and health status*.

*PEI, patient enablement instrument, mean score calculated as mean of answered items times 6, excluding cases that answered N/A or missing in >3 items*.

Table 4 shows that the FD group was more likely than the other two groups to report receiving an explanation on the diagnoses, nature, and expected course of illness, and having their concerns addressed. The ORD group was, in turn, more likely than the NRD group in reporting these processes of patient-centered care but the magnitude of difference was less than that observed between the FD and NRD groups.

### Relationships between processes and outcomes of the consultation

Table 5 shows the relationships between patient-centered process indicators and PRO of consultations. Significant positive relationships were found between each patient-centered process indicator and the PEI score, as well as other PRO of the consultation in both unadjusted and adjusted analyses. Having concerns addressed was the process indicator that had the highest correlation with all PRO indicators.

## Discussion

Our result revealed that 60% of subjects had a regular primary care doctor and among them, 60% considered their primary care providers as FDs. It was interesting to find that the public was able to make a judgment on whether their primary care doctor was a FD by function rather than qualifications. Another population survey in HK showed that the population had a good understanding of the FD concept (40). They could identify the characteristics of a FD as being one who provides accessible, comprehensive, continuous, holistic, and person-oriented care although the doctor might not have necessarily be a specialist in family medicine or have a higher qualification in family medicine. Most of the subjects with FD consulted their usual primary care doctors in the last consultation, validating the continuity of care concept that was associated with improvements in health outcomes (41–43).

Subjects with NRD were older and were more likely to have lower income than those with a FD. This finding echoed the results from another study that the elderly and those of lower income considered that having a FD was luxurious (44, 45). Notably, among those people reported not having a regular primary care doctor, one in four said they had seen their usual primary care doctor in the last consultation. It was possible that subjects with NRD might, in fact, also have their preferred doctors, although they might not always consult them first for their health problems. On the other hand, the general health perceived by subjects with ORD was much worse than that perceived by other groups. A higher percentage of them reported having chronic illnesses and requiring long-term medications compared to the other groups. It was possible that these subjects needed regular follow-up by specialists for their chronic illnesses; but they would not consult the doctor for all their health problems and thus did not consider them as FD.

In our study, we asked the subjects to rate their actual experience in the last consultation using various PRO indicators to reflect of the quality of primary care consultations, which would be more reliable than general opinions. We found that the FD group had the best PRO results, including highest mean PEI score, highest proportion of subjects being more enabled and satisfied, having improvement in health and recommending their doctors to others. Their mean PEI score (3.33) was comparable to those (3.2–4.5) found in the UK where the primary care system is uniform and every citizen has a regular general practitioner with postgraduate training (14, 24, 46, 47). On the other hand, the mean PEI score of subjects with ORD (2.63) was lower than the UK mean and it was no better than that of those with NRD. It could be possible that subjects with ORD had poorer health, which made them feel less enabled. Conversely, it could also indicate that FDs were more capable of enabling their patients than other primary care doctors. This postulation was supported by our finding that subjects with FD were more likely to receive an explanation of the illness and have their concerns addressed in the consultation, which had been associated with enablement, satisfaction, and less symptom burden (11, 42, 48–50). The importance of patient-centered practice, a core value of family medicine, was further reinforced by its significant positive relationship with PRO observed in our study. Lo et al. also found a positive association between family medicine and quality process of consultation in that primary care doctors with family medicine training prescribed fewer drugs, antibiotics, and benzodiazepines (51, 52).

Although more subjects in the FD group reported global improvement in health (OR = 1.40) and were more satisfied (OR = 2.00) after the last primary care consultation compared to NRD group, the differences between the FD and ORD groups was not significant. Our results suggested that patient enablement was a more sensitive indicator than global change in health condition or overall patient satisfaction in differentiating the quality of care between FDs and other primary care doctors. Patient enablement stresses patient empowerment, and the patient’s ability to understand and cope with his/her own health and illness (20, 22, 23), therefore, the PEI is more able and appropriate to capture the effects of patient-centered care specific to family medicine. On the other hand, the GRS is designed to detect changes in health status that is more related to the illness factor (27, 28) and overall patient satisfaction is dependent on multiple aspects of the delivery of care (23, 32). By asking whether the subject would recommend their doctors to others, we used this as an indicator of the degree of confidence in the quality of care of the doctor, which supplemented the overall satisfaction rating. This is particularly practical in a free market, pluralistic primary care system in which lay referral is an important factor for choosing a particular doctor (40). Among the three groups, subjects with FD were most likely to recommend their doctors to their family and friends while those with NRD were least likely. The results could be taken as evidence that the value of continuity of care and the establishment of a trusting patient–doctor relationship by FDs were appreciated by patients.

We believe our results could be generalized to the whole population of HK since a substantial sample size had been achieved and the sample was relatively similar in socio-demographic distribution of the local general population (53). The results might also give some insight to the process and outcomes of primary care consultations in other Asian populations including Japan, Singapore, Malaysia, and Taiwan, which have a similar primary care system as HK.

### Limitations

The definition of FD and subsequent classification into the three primary care doctor choice groups were based on self-reporting and the accuracy of the classification was not verified. The results might also be subject to retrospective recall bias of the experience of the last consultation. Since our analyses were only based on cross-sectional data, a cause and effect relationship between the choice of primary care providers and the perceived outcomes of their consultations could not be established. Finally, patient-reported process of care was not validated by any objective observation on consulting styles and skills (50, 54), empathy (55, 56), and other attributes of the practices. Further prospective studies that include direct observation of the consultation process and follow-up on outcomes are warranted to validate the quality and effectiveness of primary care consultations by different providers.

## Conclusion

Our study showed that people with a regular FD were more likely to feel enabled and to have experienced patient-centered process of care in primary care consultations, compared with people having other types of regular primary care doctors or no regular primary care doctor. They were also more likely than the others to recommend their doctors to families and friends. Patient-centered processes of care, which constituted the core of family medicine, were associated with better patient-reported outcomes. Primary care doctors should be more mindful in explaining the diagnosis, nature, and expected course of the illness, and addressing the patient’s concerns in the process of the consultation. Health policy makers should support a FD led primary care system.

## Author Contributions

Cindy L. K. Lam, Yvonne Y. C. Lo, Esther Y. T. Yu, and Carlos K. H. Wong drafted the manuscript. Cindy L. K. Lam, Yvonne Y. C. Lo, and Daniel Y. T. Fong participated in the design and implementation of the study. Carlos K. H. Wong and Daniel Y. T. Fong performed the statistical analysis. All authors read and approved the final manuscript.

## Conflict of Interest Statement

The authors declare that the research was conducted in the absence of any commercial or financial relationships that could be construed as a potential conflict of interest.

## Supplementary Material

The Supplementary Material for this article can be found online at http://www.frontiersin.org/Journal/10.3389/fmed.2014.00029/abstract

## Abbreviations

FD, family doctor; GRS, global rating scale; NRD, no regular primary care doctor; ORD, other types of regular primary care doctor; PEI, patient enablement instrument; PRO, patient-reported outcomes.
