# Supplementary material for: Having a Family Doctor is Associated with Some Better Patient-Reported Outcomes of Primary Care Consultations
Source: Front Med (Lausanne). 2014 Sep 15;1:29. doi: 10.3389/fmed.2014.00029 (PMC4292188; doi:10.3389/fmed.2014.00029)
Supplement: Supplementary file 1 [file Data_Sheet_1.PDF]

Appendix A

Questions on process and outcome measures of patient-centredness in the last consultation

Primary care consultation		
1.	Do you have a regular primary care doctor? A primary care doctor is a doctor whom you would first consult when you need to.	1. Yes (please proceed to answer 2a)<br>2. No (please proceed to answer 2b)<br>3. Not sure (please proceed to answer 2b)
2.	a. Is your regular primary care doctor a family doctor? A family doctor is a doctor whom you would consult for all types of health problems.	1. Yes<br>2. No, he/she is other types of general practitioners<br>3. No, he/she is a specialist in _______________<br>4. No, he/she is a traditional Chinese medicine practitioner<br>5. Not sure
	b. Do you have a family doctor? A family doctor is a doctor whom you would consult for all types of health problems.	1. Yes<br>2. No<br>3. Not sure
**Experience of the Last Consultation**<br>**Please refer to your last consultation for the following questions**		
3.	How long ago was your last consultation?	_________ weeks ago<br>(No=0 Not sure= -1)

	**Please ✓ one answer for each row**	**1.Yes**	**2.No**	**3.Not sure**
4.	Was the doctor your usual primary care doctor?			
5.	Did you get the following in the last consultation?			
	a. Investigation			
	b. Referral to another doctor			
	c. An explanation on the diagnosis of your illness			
	d. An explanation on the nature of your illness			
	e. An explanation on the expected course of illness			
	f. Reassurance for your concerns			
	g. Advice on self-care			
	h. Non-drug treatment including physiotherapy			
	i. Screening for diseases that you did not consult for			
	j. Life style advice, e.g. diet, exercise, smoking, drinking			

Please refer to your last consultation for the following questions:

6. How would you rate the global change in your health condition as a result of the doctor consultation?

Much better	Better	A little better	Same	A little worse	Worse	Much worse
□	□	□	□	□	□	□

7. As a result of your visit to the doctor, you are… *(Please ✓ the answer that best describes your situation)*

	Much better	Better	Same	Less	Not applicable
a. Able to cope with life	☐	☐	☐	☐	☐
b. Able to understand your illness	☐	☐	☐	☐	☐
c. Able to cope with your illness	☐	☐	☐	☐	☐
d. Able to keep yourself healthy	☐	☐	☐	☐	☐
e. Confident about your health	☐	☐	☐	☐	☐
f. Able to help yourself	☐	☐	☐	☐	☐

8. Overall, how satisfied were you with the consultation?

Very saitsfied	Quite satisfied	A little satisfied	Quite dissatisfied	Very dissatisfied
☐	☐	☐	☐	☐

9. Would you recommend this doctor to your family and friends?

Definitely yes	Maybe yes	Not sure	May be not	Definitely not
☐	☐	☐	☐	☐
